# From screening to treatment: *in vitro* and *in vivo* efficacy of phage-meropenem combination against highly virulent Carbapenem-resistant *Acinetobacter baumannii*

**DOI:** 10.1128/spectrum.03962-25

**Published:** 2026-06-15

**Authors:** Lan Liao, Shanchen Yang, Gang Luo, Ruidong Wu, Rongjing Lu, Yushan Huang, Guanfeng Wu, Wei Yi, Zhu Zeng, Zhilang Qiu, Jian Peng

**Affiliations:** 1 Guizhou Key Laboratory of Microbio and Infectious Diseases Prevention & Control, School of Biology and Engineering (School of Modern Industry for Health and medicine)/School of Basic Medical Sciences, Guizhou Medical University 74628, Guiyang, Guizhou, China; 2 Engineering Research Center for Bio-Perception Materials of Guizhou Province, Guiyang, China; 3 Engineering Research Center of Health Medicine Biotechnology of Institution of Higher Education of Guizhou Province, Guizhou Medical University 74628, Guiyang, Guizhou, China; 4 Department of Cardiovascular Surgery, Affiliated Hospital of Zunyi Medical University 364187, Guiyang, Guizhou, China; Second Affiliated Hospital of Soochow University, Suzhou, China

**Keywords:** CRAB, virulence, phage therapy, quorum sensing

## Abstract

Carbapenem-resistant *Acinetobacter baumannii* (CRAB) is a hospital-acquired pathogen of significant clinical importance. Given the slow progress in antibiotic development, phages have emerged as a novel therapeutic strategy against CRAB infections due to their synergistic effects with antibiotics. This study aims to explore phage-antibiotic combination therapy targeting CRAB, thereby providing a theoretical foundation for its clinical translation. Through virulence assessments in *Galleria mellonella* larvae and mice, CRAB110 was identified as the most virulent strain among 199 clinical CRAB isolates. Transcription analysis revealed that its enhanced pathogenicity may be associated with dysregulated pro-inflammatory responses. Using CRAB110 as the host bacterium, the newly isolated phage, P2, was selected for further study due to its high lytic efficiency and broad host range. The CRAB110 mutant strain CRAB110-2N, treated with P2, exhibited increased drug susceptibility and attenuated virulence compared to the wild-type strain. Both *in vitro* and *in vivo* experiments demonstrated that the combination of P2 with antibiotics achieved superior bacterial clearance compared to phage therapy alone. Furthermore, whole-genome analysis of CRAB110-2N revealed a mutation in its *ugd*. The constructed CRAB110Δ*ugd* showed tolerance to P2; the quorum-sensing gene *abaI* was associated with phage susceptibility, with the CRAB110Δ*abaI* strain displaying insensitivity to P2. These findings provide experimental evidence supporting the clinical application of phage-antibiotic combination therapy.

**IMPORTANCE:**

This study focused on phage-antibiotic combination therapy targeting carbapenem-resistant *Acinetobacter baumannii* (CRAB), a priority pathogen designated by the World Health Organization (WHO). A highly virulent strain, CRAB110, was screened from 199 clinical isolates, and a novel, highly lytic phage, P2, was isolated from sewage samples. CRAB110-2N, a mutant strain induced by P2 exposure, displayed increased antibiotic susceptibility and attenuated virulence. Key findings showed that the combination of P2 and meropenem achieved a 99% bacterial clearance rate *in vitro* and exerted significant synergistic effects *in vivo*. Genomic analysis identified mutations in the *ugd* gene, and knockout experiments confirmed its correlation with phage susceptibility. Additionally, the quorum-sensing gene abaI was found to be associated with phage susceptibility in CRAB. Collectively, these results provide critical experimental evidence for the clinical translation of this phage-antibiotic combination therapy.

## INTRODUCTION

*Acinetobacter baumannii* (*A. baumannii*)*,* a pathogen endemic to both healthcare facilities and community settings, has been classified as a critical-priority multidrug-resistant organism by global health authorities (1, 2). *A. baumannii* is associated with a diverse range of clinical manifestations, including pneumonia, bloodstream infections, soft tissue infections, urinary tract infections, and sepsis (3–5). Surveillance data indicate that during the peak of the COVID-19 pandemic, the nosocomial transmission rate reached a maximum of 78% (6). This contributes to an estimated annual global burden of 2 million infections and 450,000 attributable fatalities. Without urgent intervention to control antimicrobial resistance (AMR), multidrug-resistant pathogens are projected to become a leading cause of global mortality. Conservative modeling suggests that cumulative fatalities due to bacterial AMR could reach approximately 10 million annually by 2050 (7, 8). Driven by the misuse of antimicrobials, carbapenem-resistant *A. baumannii* (CRAB) is experiencing an accelerating global spread. In its latest 2024 surveillance update, the World Health Organization (WHO) has designated CRAB as a critical priority pathogen, coinciding with an increasing clinical recognition of hypervirulent multidrug-resistant phenotypes. Anna C. Jacobs et al. have characterized the clinically isolated AB5075, a multidrug-resistant hypervirulent variant of *A. baumannii*. The synergistic interaction of its complex resistance determinants and virulence-associated macromolecules includes porin modifications, structural alterations in lipopolysaccharide, and capsular polysaccharide biosynthesis systems (9, 10). This significantly exacerbates the therapeutic challenges associated with nosocomial infections. Notably, this convergent resistance-virulence phenotype exhibits epidemiological similarities with other critical-priority pathogens, particularly in hypervirulent carbapenem-resistant *Klebsiella pneumoniae* clones (11). Therefore, the development of non-antibiotic therapeutic modalities is an urgent necessity in contemporary antimicrobial stewardship to address the existential threat posed by multidrug-resistant pathogens.

Contemporary therapeutic strategies for combating multidrug-resistant bacterial infections primarily encompass three modalities: next-generation antimicrobial agents, engineered antimicrobial peptides, and precisely calibrated phage interventions (12–15). Although conventional antibiotics remain the primary therapeutic options for bacterial infections, the rapid evolutionary trajectories of pathogens, coupled with the prolonged drug development processes, often result in antimicrobial innovation lagging behind microbial adaptation (16, 17). In contrast, engineered antimicrobial peptides offer rapid development pathways, thereby addressing this therapeutic gap and exhibiting potent bactericidal effects against multidrug-resistant phenotypes. Preclinical studies have shown that the engineered peptides dN4 and dC4 demonstrate therapeutic efficacy in murine infection models (18). Peptide LI14 demonstrates a synergistic enhancement when used in conjunction with conventional antibiotics and exhibits robust activity against multidrug-resistant clinical isolates (19). Despite their therapeutic potential, most antimicrobial peptides face significant pharmacological limitations, including suboptimal *in vivo* stability and dose-limiting cytotoxicity toward host cells and commensal microbiota. Phage therapy overcomes these inherent limitations through evolutionarily refined targeting mechanisms.

Phage therapeutics have emerged as a crucial frontier in the battle against multidrug-resistant pathogens, leveraging evolutionarily optimized precision lytic activity (20). Remarkable biosafety profiles are characterized by minimal off-target cytotoxicity (21, 22), cost-effective scalable production (23), and an unprecedented phylogenetic diversity exceeding 10^31^ virions globally (7). As early as 1926, d'Hérelle utilized phages for therapeutic intervention in patients suffering from malaria, plague, cholera, and other infectious diseases. Similarly, in 1958, Professor Yu Jian in China achieved successful therapeutic outcomes by employing phages to treat infections caused by *Pseudomonas aeruginosa* in clinical settings. In 2025, the WHO formally recognized the therapeutic potential of phage therapy for antibiotic-resistant bacterial infections, prompting countries, such as Belgium, China, Portugal, and the United Kingdom, to subsequently issue official clinical guidelines governing its application (24). An increasing body of evidence indicates that phage treatment can effectively reverse antimicrobial resistance and mitigate virulence factors in bacterial pathogens (7, 25, 26). Notably, the therapeutic efficacy of phage interventions against multidrug-resistant *Pseudomonas aeruginosa* infections in cystic fibrosis patients has been documented to restore susceptibility to imipenem while simultaneously reducing endotoxin release (25).

In recent years, significant research efforts have been devoted toward investigating the interaction between phages and bacterial quorum sensing (QS) to refine phage therapy regimens further (27–30). QS serves as a widespread mechanism for intercellular communication among bacteria, modulating various collective behaviors, such as biofilm formation, expression of virulence factors, antibiotic resistance, and metabolic adaptation, all of which depend on population density (29, 31–33). In *A. baumannii*, QS is primarily regulated by the genes *abaI* and *abaR*. The *abaI* encodes an acyl-homoserine lactone (AHL) synthase, which is responsible for synthesizing N-acylhomoserine lactone signal molecules, thereby establishing the molecular basis for QS signal transduction. Concurrently, the *abaR* encodes the AHL receptor protein, AbaR (33–37). When the AHL signals accumulate beyond a threshold concentration, the AbaR protein binds to these AHL molecules, functioning as a transcriptional regulator. This interaction can either activate or repress the expression of target genes (38).

The issue of *A. baumannii* antibiotic resistance is becoming increasingly severe, with its virulence continuously escalating, significantly complicating treatment. To address this problem, this study conducted virulence assessments on 199 previously preserved clinical CRAB strains (39). Through screening, we identified a highly virulent strain designated CRAB110. After treatment with P2 antibiotic, a P2-resistant mutant strain, CRAB110-2N, was isolated. Phenotypic analysis revealed that this resistant strain exhibited reduced antibiotic sensitivity and virulence. Both *in vivo* and *in vitro* experiments confirmed that the combination of phages and antibiotics yielded superior therapeutic effects. *Ugd* in CRAB110-2N was found to be mutated, and subsequently, a CRAB110Δ*ugd* mutant strain was constructed, demonstrating significant tolerance to P2 antibiotic. Given the close association between QS and bacterial virulence, as well as antibiotic resistance (40–42), along with existing evidence indicating interactions between QS and phage, we further investigated changes in phage sensitivity in the *abaI* mutant strain. The results demonstrate that the deletion of the QS gene *abaI* may be associated with phage sensitivity. This study provides an important theoretical foundation for phage monotherapy and phage-antibiotic combination strategies against infections.

## MATERIALS AND METHODS

### Strains, *Galleria mellonella*, and mice

This study utilized a cohort of specific pathogen-free male BALB/c mice, aged 6 weeks, with a mean body weight of 20 g. *Galleria mellonella* larvae (*G. mellonella* larvae), averaging 0.23 g in weight and measuring 2.5 cm in length, were procured from Tianjin Huiyude Biotechnology Co., Ltd. All laboratory consumables were sourced from SpiboBiotech (Beijing) Co., Ltd. (Animal License Number: SYXK [Beijing] 2023-0002), while rodent feed and bedding materials were obtained from Beijing Huanyu Zhongke Biotechnology Co., Ltd. The experimental bacterial repository comprised 199 clinically derived CRAB strains, which were cryopreserved in our institutional biobank (43).

### Screening of highly virulent CRAB isolates

#### *G. mellonella* larvae virulence assay

Virulence stratification was performed using *G. mellonella* larvae lethality assays involving 199 CRAB isolates. The top 10% of strains were selected for triplicate dose-escalation studies, with concentrations ranging from 10^7^ to 10^9^ CFU/mL in 5 μL, administered to the last left proleg (7, 44). Mortality kinetics were recorded hourly at 37°C over a period of 96 hours to establish virulence indices before conducting murine challenge experiments. Statistical analysis of the *G. mellonella* survival larvae data was conducted using the Kaplan-Meier method. Differences between groups were compared using the Log-rank test, performed with GraphPad Prism 10.0 software. A *P* value of less than 0.05 was deemed statistically significant.

#### Mouse virulence assay

In the assessment of murine pathogenicity, strains exhibiting extreme virulence in *G. mellonella* larvae were prioritized. Following dose calibration with maximally lethal isolates, anesthetized mice were subjected to an intratracheal challenge using a 20 μL suspension containing 5 × 10⁷ CFU (45). Mortality kinetics were monitored at 6-hour intervals over 7 days to determine virulence hierarchies. Survival data from murine experiments were analyzed using the Kaplan-Meier method and the Log-rank test. Statistical significance was defined as *P* < 0.05.

#### Lung transcriptomics of mice infected with high-virulence CRAB110 and low-virulence CRAB55

From a total of 199 CRAB strains, the most virulent and least virulent strains were selected for infection studies in mice. Subsequently, lung tissue RNA was extracted and sent to Shanghai Majorbio Bio-pharm Technology Co., Ltd. for library construction and sequencing analysis. The transcriptomes of CRAB110 and CRAB55 were sequenced using next-generation sequencing technology to compare their differential expression profiles. The specific steps are outlined as follows: first, RNA was extracted from CRAB110 and CRAB55, and the integrity and concentration of the RNA were assessed using the Agilent 2100 Bioanalyzer. After passing quality control, the mRNA was randomly fragmented, and libraries were constructed using a strand-specific library construction method. Upon completion of the library construction, preliminary quantification was performed using the Qubit 2.0 Fluorometer, followed by an assessment of the library insert fragment size using the Agilent 2100 Bioanalyzer. The qualified libraries were then pooled for Illumina sequencing. Subsequently, gene expression quantification analysis, along with Gene Ontology (GO) and Kyoto Encyclopedia of Genes and Genomes (KEGG) enrichment analyses, was conducted on the sequencing data. For transcriptomic analysis, differential gene expression between CRAB110 and CRAB55 was identified using the DESeq2 package (version 1.38.3) in R (46). Genes with an absolute log2 fold change greater than 2, and an adjusted *P* value (false discovery rate, FDR) of less than 0.01 were considered significantly differentially expressed. GO functional and KEGG pathway enrichment analyses of the differentially expressed genes were performed using the cluster Profiler R package (version 4.6.2) (47). The significance of enrichment was assessed using a hypergeometric test, with *P* values adjusted for multiple comparisons using the Benjamini-Hochberg method. An adjusted *P* value (FDR) of less than 0.01 was defined as statistically significant for enrichment.

### Screening of phages targeting CRAB

#### Phage isolation

Wastewater samples were collected from a tertiary hospital and its associated sewage treatment plant in Guiyang, Guizhou, China. Following initial clarification through centrifugation at 10,000 rpm for 10 minutes, the supernatants were subjected to sterile filtration using a 0.22 µm pore size filter (7). The processed filtrates were incubated with the host bacteria for 10 minutes before being transferred onto double-agar layer plates. Subsequently, the plates were incubated to facilitate phage propagation and plaque formation (48). The isolated and purified phages were designated as phage 1 (P1), phage 2 (P2), phage 3 (P3), and so forth.

#### Assessment of phage lytic kinetics

An overnight culture of CRAB110 was harvested during the exponential growth phase, washed in sterile saline, and standardized to a concentration of 1 × 10⁶ CFU/mL. In sterile 96-well microtiter plates, 150 μL of LB broth was dispensed, followed by the sequential addition of 40 μL of bacterial suspension and 10 μL of purified phage lysates (P1–P4). The final reaction volume of 200 μL achieved a predefined multiplicity of infection. Bacteriolytic activity was quantified kinetically by monitoring the optical density at 600 nm (OD_600_) at 10-minute intervals over a 24-hour period using a BioTek LogPhase 600 spectrophotometer (Agilent), thereby generating phage-specific antibacterial activity profiles.

#### Host range profiling of phages

A diverse set of 42 *A. baumannii* isolates (10 MLST-KL types) was standardized to the exponential phase (OD₆₀₀ = 0.5) following overnight culture. For host range analysis, bacterial suspensions in molten soft agar (10 mL, 55°C) were overlaid onto LB agar plates after thorough homogenization. A P2 lysate (10 μL, 1 × 10⁸ PFU/mL) was then spotted onto the solidified overlay. Lytic competence was assessed based on the formation of zones of clearance after 18 to 24 hours of incubation (49).

#### Transmission electron microscopy of P2

Purified phage suspensions were adsorbed onto carbon-coated copper grids (300 mesh) and fixed with 3% (vol:vol) glutaraldehyde at 4°C for 3 minutes. The grids were air-dried, negatively stained with 2% (wt/vol) phosphotungstic acid for 30 seconds, and subsequently desiccated before examination (7).

### Biological characterization of P2

#### P2 genome analysis

Purified virions were subjected to enzymatic treatment using DNase I and RNase I at 37°C for 2 hours, followed by 0.22 μm filtration to eliminate exogenous nucleic acids. Nucleic acid extraction was conducted utilizing a virus DNA/RNA kit (DP315), after which the samples were sent to Sangon Biotech for whole-genome sequencing.

The complete genome sequence of the phage should be submitted to the NCBI database, followed by online homology alignment using the BLASTN algorithm. Based on the alignment results, the 20 phages exhibiting the highest sequence similarity to P2 should be selected, and their complete genome sequence files must be downloaded from the NCBI GenBank database for subsequent analysis. Furthermore, the online analysis tool VICTOR (https://viridic.icbm.de) (50), which is based on Viral Proteomic Homology, should be utilized to conduct systematic pairwise comparisons of the genomes of P2 and the aforementioned phages. This platform generates a homology matrix by calculating the global average amino acid identity (AAI) between genomic protein sequences. In this analysis, the 95% AAI threshold recommended by the International Committee on Taxonomy of Viruses is adopted as the primary molecular criterion for determining whether phages belong to the same species. If the highest homology score with all known phage sequences remains below the 95% species threshold, it indicates that the phage is not currently recorded at the species level within existing databases and classification frameworks, and can thus be preliminarily identified as a novel phage (51).

Upon receiving the sequencing results, open reading frames and genomic functions were predicted using PHASTEST (https://phastest.ca), supplemented by additional predictions from RAST. Phylogenetic trees were constructed using VipTree (https://www.genome.jp/viptree), followed by a comparative genomic analysis against the most closely related phages.

#### Determination of the MOI and stability analysis of P2

The multiplicity of infection (MOI) refers to the ratio of phages to bacteria. To determine the optimal MOI, we assessed values of 0.01, 0.1, 1, 10, and 100. This process involved mixing phage suspensions at a concentration of 1 × 10^6^ PFU/mL with various bacterial strains, followed by the measurement of progeny phage titers on double-layer plates.

P2 was mixed with CRAB110 in a 1:1 (vol:vol) ratio, resulting in an MOI of 0.01, followed by a 1-minute incubation period. The mixture was centrifuged at 8,000 rpm for 1 minute, followed by a second centrifugation at 10,000 rpm for 1 minute to collect the sediment. The sediment was washed twice and then resuspended in 20 mL of LB medium. Samples were collected at 10-minute intervals to determine phage titers, and the one-step growth curve for P2 was subsequently plotted (52, 53).

The effects of pH, temperature, and chloroform on phage activity were investigated. Phages were treated at temperatures of 10°C, 20°C, 30°C, 40°C, 50°C, and 60°C for 30 minutes, followed by a 10-minute mixing period with CRAB110 before assessing phage titer. The phage suspensions were adjusted to pH values ranging from 4 to 10, incubated at room temperature for 30 minutes, and subsequently subjected to titer determination. Additionally, P2 was treated with chloroform at concentrations of 0%, 1%, 3%, and 5%, after which phage titers were quantified.

### Biological characteristics study of phage-resistant bacteria

In conducting drug susceptibility testing on resistant strains, the 96-well plate method was employed, utilizing a reaction system of 200 μL per well. The final concentrations of meropenem (MER), chloramphenicol, lincomycin, and vancomycin were adjusted to 128, 64, 32, 16, 8, and 4 μg/mL, respectively. Subsequently, a bacterial suspension was added to achieve a final bacterial concentration of 5 × 10^5^ CFU/mL. After 18 hours of incubation, the OD_600_ value was measured. An OD_600_ value, after subtracting the blank medium, of less than 0.05 indicated no bacterial growth, representing the minimum inhibitory concentration.

To evaluate the virulence of resistant bacteria, we conducted animal experiments. First, 5 μL of a bacterial suspension with a concentration of 1 × 10^9^ CFU/mL was injected into the greater wax moth, with observations and recordings made every 6 hours. Simultaneously, 100 μL of a bacterial suspension at a concentration of 2.5 × 10^8^ CFU/mL was intraperitoneally injected into mice, with observations and recordings made every 24 hours.

### P2 Combined with MER to eliminate CRAB110

#### Combined with MER to eliminate CRAB110 *in vitro*

P2 titers, ranging from 1 × 10^7^ to 1 × 10^1^ PFU/mL, were paired with MER concentrations from 128 to 2 μg/mL. Following the addition of 100 μL of CRAB110 suspension (1 × 10⁶ CFU/mL) per well, the total volume was adjusted to 200 μL. Antibacterial activity was quantified using the BioTek Log Phase 600 (Agilent), with the optimal combination’s clearance rate calculated using the formula w = (A − B)/A, where A represents the phage/antibiotic-free control and B represents the phage-antibiotic mixture.

#### Combined with MER to eliminate CRAB110 *in vivo*

Inject 100 μL of CRAB110 bacterial suspension at a concentration of 2.5 × 10^8^ CFU/mL into the right side of the mouse’s abdominal cavity. Thirty minutes post-infection, administer the treatments via injection into the left side of the abdominal cavity. The treatment groups consist of three categories: one receiving 20 mg/kg of MER, another receiving P2, and a third receiving a combination of MER and phage. The CRAB110 injected group serves as the control, with 10 mice allocated to each group. Subsequently, monitor and document the survival status of the mice every 24 hours until the conclusion of the experiment. Mouse survival data were analyzed using the Kaplan-Meier method, and pairwise comparisons between treatment groups were conducted using the Log-rank test. A *P* value of less than 0.05 was considered statistically significant.

### Safety assessment of P2

To systematically evaluate the biosafety profile of P2, *G. mellonella* larvae (*n* = 10 per group) were subjected to microinjections of 5 μL of phage suspension (1 × 10^8^ PFU/mL). Survival kinetics were documented at 6-hour intervals over a period of 48 hours across biological triplicates. In parallel, murine studies involved the intraperitoneal administration of 100 μL of phage suspension to cohorts (*n* = 10 per group). Rodent survival was quantitatively assessed daily for 8 days. Survival curves for both *G. mellonella* larvae and mice in the safety assessment were constructed using the Kaplan-Meier method. These curves were then compared to the PBS control groups through the application of the Log-rank test.

### The CPS synthesis gene *ugd* mediates CRAB susceptibility to P2

A resistant strain, designated CRAB110-2N, was isolated. Following three rounds of purification, genomic DNA was extracted and subsequently submitted to Sangon Biotech for whole-genome sequencing utilizing an Illumina next-generation sequencing platform. The assemblies were generated using SOAP denovo (v2.04) (54, 55), SPAdes (v3.13.1) (56), and ABySS (v2.2.4) (57), followed by optimization through gap closure using Gap Closer (v1.12) (58). After filtering out contigs shorter than 500 bp, the complete genome sequence was compared with that of the wild-type strain using NCBI tools for mutation identification.

The differentially expressed gene *ugd* was selected for the construction of the mutant strain CRAB110Δ*ugd*. Subsequently, the CRAB110Δ*ugd* strain was cultured overnight, and 100 μL of the bacterial suspension was mixed with 10 mL of semi-solid medium and poured onto plates. After the medium solidified, 10 μL of P2 was spotted onto the plates, which were then air-dried and incubated upside-down overnight to assess lytic activity. Meanwhile, the bacterial concentrations of both CRAB110 wild type and CRAB110Δ*ugd* were adjusted to 1 × 10^8^ CFU/mL. In a 96-well plate, 150 μL of LB broth was mixed with 30 μL of bacterial suspension and 20 μL of phage solution. Bactericidal activity was monitored hourly for 24 hours using the BioTek LogPhase 600 (Agilent).

### Effects of quorum-sensing gene *abaI* on phage susceptibility

The sensitivity of *abaI* to P2 was investigated using the method described in section “The CPS synthesis gene *ugd* mediates CRAB susceptibility to P2.”

## RESULTS

### Identification of CRAB with elevated virulence

#### Virulence assessment of bacterial strains

**Fig 1:**
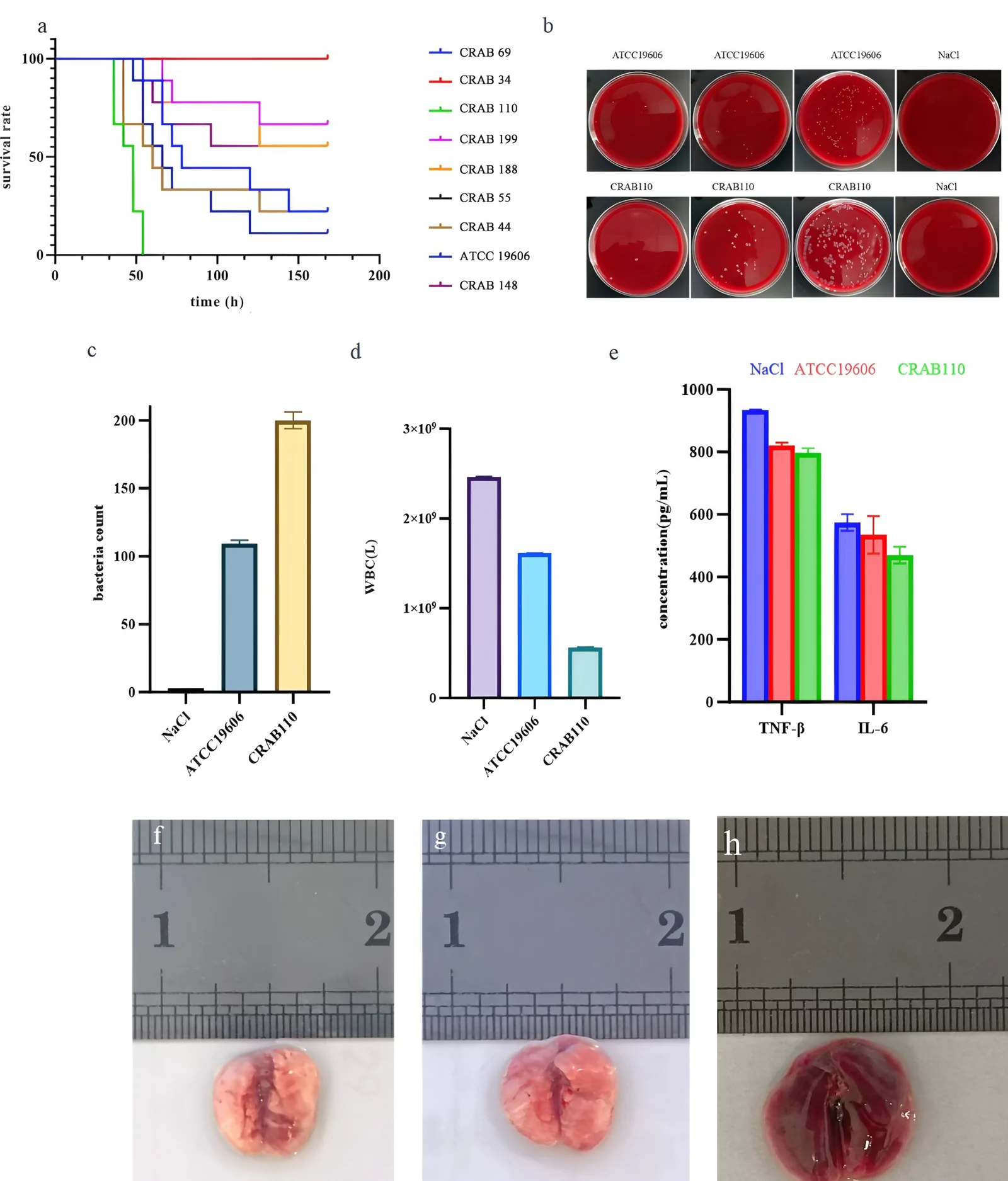
The mortality rate of mice following bacterial infection, alongside the detection of bacterial counts in the lungs, levels of white blood cells (WBC), and inflammatory factors. (**a**) Mortality rates in mice post-lung infection with various strains are presented. All mice infected with CRAB110 succumbed to the infection within 54 hours post-infection, whereas no mortality was observed in those infected with CRAB55 (*P < 0.05). (**b**) Results obtained from homogenate dilution, followed by the coating culture of lung tissue are shown. (**c**) Quantification of bacterial loads following dilution and plating of lung tissue homogenates indicates that the bacterial count for CRAB110 was the highest (****P < 0.0001). (**d**) WBC results for infected strains at 1 hour post-infection reveal that the WBC count was lowest in the CRAB110-infected group (****P < 0.0001). (**e**) Concentrations of interleukin-6 (IL-6) and tumor necrosis factor-beta (TNF-β) in serum 1 hour after infection demonstrate that the production of inflammatory factors was lowest following CRAB110 infection (****P < 0.0001). Representative pulmonary dissection specimens from murine models are depicted in (**f**) isotonic saline control, (**g**) cyclophosphamide-induced immunosuppression with saline instillation, and (**h**) cyclophosphamide-induced immunosuppression, followed by CRAB110 infection.

Among the 199 CRAB isolates preserved in our laboratory, the virulence of these strains was evaluated using *G. mellonella* larvae. The results indicated that the CRAB55 did not induce any mortality, whereas the CRAB44 and CRAB148 led to a mortality rate of 10%. In contrast, the CRAB34, CRAB69, CRAB110, CRAB186, CRAB188, and CRAB199 exhibited mortality rates exceeding 90% within 24 hours (Table S1). The overall distribution of mortality rates across different ranges was approximately as follows: 0%–20% (4.9%), 30%–40% (10.3%), 50%–60% (15.8%), 70%–80% (37.4%), and 90%–100% (31.5%). Subsequently, five strains that caused 100% mortality at 96 hours and three strains with mortality rates below 10% were selected for murine virulence assays. The results demonstrated that infection with the CRAB110 resulted in 100% mortality in mice within 54 hours. The mortality rates of the other tested strains, ranked in descending order of virulence, were ATCC19606, CRAB44, CRAB69, CRAB188, CRAB148, CRAB199, CRAB34, and CRAB55 (Fig. 1a), confirming CRAB110 as the most virulent strain. The survival differences among all groups were statistically significant (*P* < 0.05).

Mice were infected via intratracheal injection with bacterial suspensions of ATCC19606 and CRAB110. At 24 hours post-infection, lung tissues were excised and homogenized (10% wt/vol). Serial dilutions of the homogenates were inoculated onto Columbia blood agar (Fig. 1b). Considerable bacterial growth was observed for both ATCC19606 and CRAB110 (plated at dilutions of 10⁶, 10⁵, 10⁴), in contrast to the saline control group, which exhibited no growth. Bacterial enumeration (Fig. 1c) showed statistically significant differences between the infection groups (*P* < 0.05). The bacterial concentrations in lung tissue at 24 hours post-infection were approximately 8.36 × 10⁷ CFU/g for CRAB110 and 5.58 × 10⁷ CFU/g for ATCC19606, confirming a significantly higher bacterial load in the CRAB110-infected group.

An assessment of white blood cells (WBC), interleukin-6 (IL-6), tumor necrosis factor-beta (TNF-β), and clinical signs was conducted to verify the immunosuppressive effects of cyclophosphamide in mice. Blood samples were collected for complete blood count analysis 3 days following the intraperitoneal injection. The results demonstrated significant differences in WBC levels between the cyclophosphamide-injected group and the saline group (*P <* 0.05). This finding indicates adequate immunosuppression, which is crucial for establishing the pneumonia model in mice. Compared to pre-infection levels, WBC counts measured 1 hour post-lung infection showed a decline (Fig. 1d), suggesting that cyclophosphamide exerted persistent immunosuppressive effects following infection. WBC counts in the ATCC19606 infection group were approximately three times higher than those in the CRAB110 infection group, yet remained lower than in the saline group. This indicates a differential influence of bacterial strain infection on the intensity of the mouse immune response. The observed WBC counts in the infected groups served as an index of the extent of immune regulatory system impairment. Notably, the CRAB110 infection group exhibited the lowest WBC count, further corroborating the greater virulence of CRAB110 compared to ATCC19606. Analysis of serum IL-6 and TNF-β levels 1 hour post-infection demonstrated significant decreases in both cytokines within the infected groups compared to the negative control group, with the CRAB110 infection group showing lower values than the ATCC19606 group (Fig. 1e), indicating that CRAB110 induced more severe impairment of the immune system.

During the entire infection monitoring period in mice, notable morphological disparities were observed between the infected and saline groups (Fig. S1). Necropsy of the experimental cohorts at 54 hours post-intervention revealed that both the saline control group and the cyclophosphamide-immunosuppressed plus saline group exhibited uniformly pale-pink pulmonary parenchyma with well-preserved architecture, showing no signs of macroscopic pathological changes (Fig. 1f through g). In contrast, the lungs of the cyclophosphamide-immunosuppressed + CRAB110 infected group exhibited diffuse dark-red discoloration accompanied by significant congestion (Fig. 1h), which aligns with the pathognomonic characteristics of CRAB110-induced pneumonic consolidation. Scanning electron microscopy observations of the lungs in the saline group and the CRAB110 infection group revealed that the CRAB110 infection group exhibited a significant presence of bacteria in the lungs, whereas the cyclophosphamide group did not show any bacterial formation (Fig. S2).

#### Lung transcriptomics of mice infected with high-virulence CRAB110 and low-virulence CRAB55

**Fig 2:**
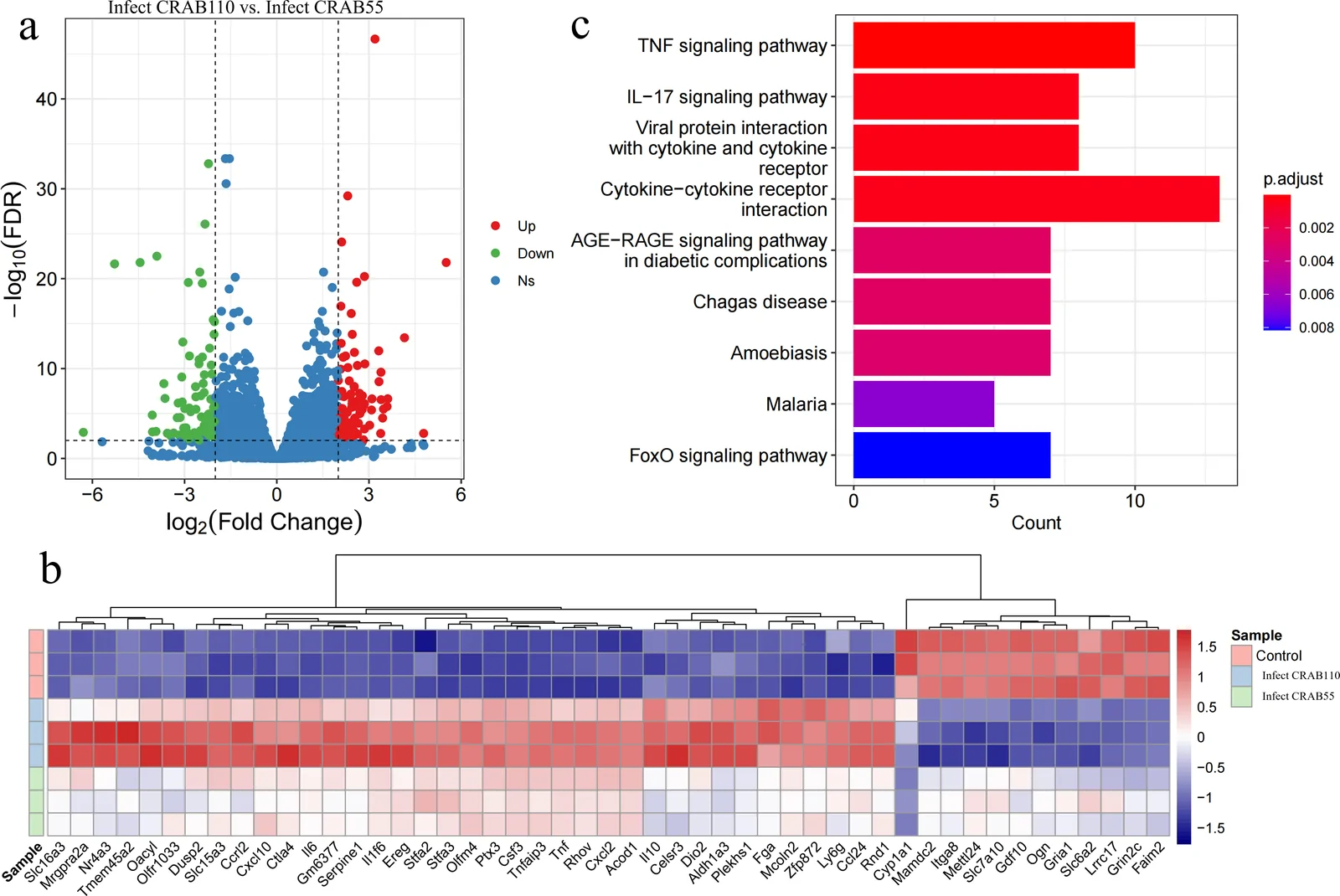
Transcriptomic analysis of lung tissue from mice infected with CRAB110 in comparison to those infected with CRAB55. (**a**) This section compares the gene expression profiles between mice infected with CRAB110 and those infected with CRAB55. The plot illustrates the significance level of differential expression in relation to the fold change in gene expression (log₂ Fold Change). Each point on the plot represents an individual gene, with green and red points indicating down-regulated and up-regulated differentially expressed genes, respectively; blue points signify non-differentially expressed genes. (**b**) The hierarchical clustering of differentially expressed mRNAs includes *Cyp1a1*, *lc6a2*, *amdc2*, *tga8*, and *TNF*.(**c**) The abscissa denotes the KEGG pathway, while the ordinate represents the significance level of pathway enrichment.

In a study involving 199 CRAB strains, CRAB110 exhibited the highest virulence, whereas CRAB55 demonstrated the lowest. Transcriptomic analysis of RNA extracted from the lung tissue of mice infected with these strains revealed distinct patterns in gene expression. Notable differentially expressed genes included *Cyp1a1, lc6a2, amdc2, df10, tga8,* and *TNF* (Fig. 2a). A total of 88 down-regulated genes and 93 up-regulated genes were identified (Fig. 2b). Key categories among these differentially expressed genes included immune regulatory genes (*IL1B, IL6, IL12A, IL1R2, IL23A*), inflammation-related genes (*TNF, TNFAIP3, TNFAIP6, TNIP3*), immune cell function-related genes (*CD14, CD274, CD300LF, CD33, CD80*), and chemokines (*CCL2, CCL3, CCL4, CCL7, CCL12, CCL20, CCL24, CXCL1, CXCL2, CXCL5, CXCL10*) (Table S2). KEGG enrichment analysis identified pathways associated with tumor cell signaling and cytokine signaling, including the cytokine-cytokine receptor interaction (Fig. 2c). *CCL2* and *TNF*, both pro-inflammatory cytokines, were significantly enriched in inflammatory pathways, suggesting that CRAB110 may enhance its virulence by exploiting inflammatory responses. In conclusion, having been identified as the most virulent strain among the 199 CRAB isolates, CRAB110 was selected as the target host for subsequent investigations into phages specific to this strain.

### Collection of CRAB110 phages

#### Screening of CRAB110 phages

**Fig 3:**
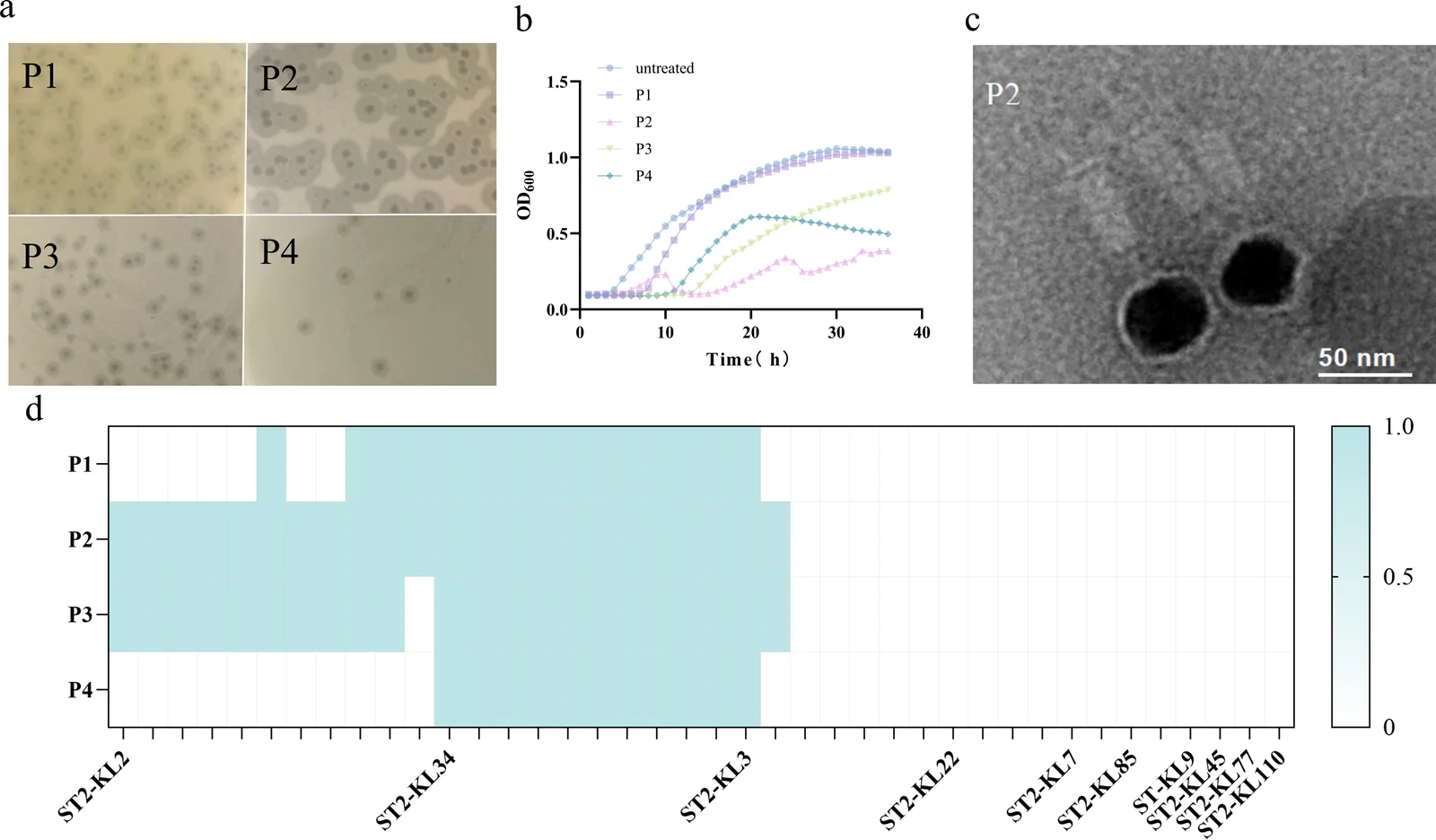
Collection of the CRAB110 phage alongside observations obtained from scanning electron microscopy. (**a**) Plaques formed by four distinct CRAB110 phages. (**b**) Inhibition curves associated with these four CRAB110 phages. (**c**) Electron microscopic image of P2. (**d**) Phage sensitivity profiles of P1–P4 against 42 CRAB strains, where blue indicates sensitivity and white denotes insensitivity.

Using CRAB110 as the host strain, phages were screened from wastewater samples collected from a tertiary hospital and a wastewater treatment plant in Guiyang. As a result, four phage strains were identified and designated as phage 1 (P1), phage 2 (P2), phage 3 (P3), and phage 4 (P4). P1 and P4 formed plaques with an approximate diameter of 1 mm, while P3 produced plaques measuring between 1 and 2 mm. Notably, P2 exhibited plaques of 3 to 4 mm with distinct halos, indicating the presence of virulent phages (Fig. 3a).

The *in vitro* antibacterial efficacy of P1–P4 was evaluated. The results demonstrated that P1 led to the emergence of resistant bacteria after 8.5 hours. P2 initially produced resistant bacteria but subsequently eradicated them, with the final optical density at 600 nm (OD_600_) remaining below 0.5. P3 resulted in the emergence of resistant bacteria by 15 hours, reaching a maximum OD_600_ of 0.784. P4 developed resistance after 12.5 hours, with an OD_600_ of 0.611. Notably, P2 exhibited the most favorable antibacterial effect among the phages tested (Fig. 3b).

The host range of phages 1–4 was evaluated using the spot test on 42 strains of *A. baumannii*. The results indicated that CRAB110 belongs to sequence type ST2-KL34. P2 exhibited sensitivity to both ST2-KL34 and the majority of ST2-KL2 strains (Fig. 3d), highlighting its broader lytic spectrum and justifying its selection for further experiments. Transmission electron microscopy revealed that P2 possesses an icosahedral head structure, with a head diameter of 47.84 nm and a tail length of 74.92 nm (Fig. 3c).

#### Biological characteristics of P2

##### Genome analysis of P2

**Fig 4:**
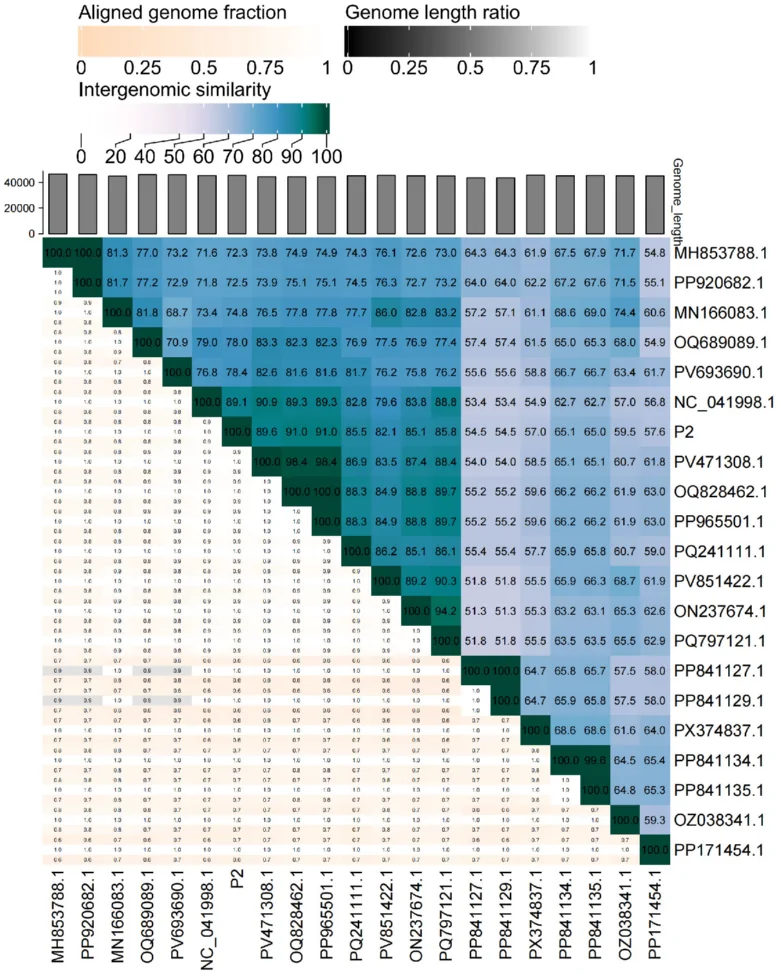
Heatmap illustrating the genomic alignment characteristics of the top 20 genomes most similar to strain P2 in the NCBI database. In the main plot area, the cell values represent the sequence similarity between corresponding genome pairs, measured as percentages ranging from 0 to 100. The intensity of the background color is positively correlated with similarity. According to the established evaluation criteria, strains exhibiting sequence similarity below 95% are classified as novel phages; therefore, strain P2 is identified as a novel phage.

After aligning the P2 sequence with the NCBI database, we downloaded the sequences of the 20 most similar phages and analyzed them using the viridic.icbm.de website. These results indicated that the highest similarity to P2 was 91% (Fig. 4), which is below the 95% threshold. This finding suggests that P2 is a novel phage. The complete genome analysis of P2 revealed a total length of 45,509 base pairs (bp) and a guanine-cytosine (GC) content of 37.74%. An evolutionary tree was constructed using the Vip Tree, indicating that P2 belongs to the category of *Acinetobacter* phages (Fig. S3). Functional annotation of P2 identified a total of 85 open reading frames, which include hypothetical proteins, regulatory proteins, structural proteins, baseplate proteins, tail fiber proteins, depolymerases, and lytic enzymes (Fig. S4).

##### Determination of Optimal Multiplicity of Infection, One-step Growth Curve, and Stability of P2

The MOI refers to the ratio of phages to bacteria. To establish the optimal MOI for P2, various MOIs were examined. Phage yields were quantified using double-layer plates. The results indicated that the maximum release of progeny phages occurred at an MOI of 0.01, achieving a concentration of 3.44 × 10^9^ PFU/mL. This finding suggests that an MOI of 0.01 is optimal for P2 (Fig. S5).

To elucidate the growth characteristics of P2, titers were measured at 10-minute intervals. The results indicated that P2 initiated lysis at 60 minutes, reaching its peak at 120 minutes, after which the phage titer stabilized, thereby drawing a one-step growth curve (Fig. S5).

To evaluate the stability of P2, we explored the effects of temperature, pH, and chloroform on phage stability. The results indicated a significant decline in phage titer at 50°C. At the same time, stability was maintained within the temperature range of 10°C–50°C (Fig. S5). Additionally, we tested the phage stability across a variety of pH levels. The findings suggested that pH values below 5 and above 9 resulted in reduced phage titers. In contrast, the phage remained stable within the pH range of 5–9, indicating that P2 possesses a relatively broad pH tolerance (Fig. S5). Furthermore, the stability of P2 was assessed in the presence of chloroform at concentrations of 0%, 1%, 3%, and 5%. The results revealed minimal influence on phage viability, confirming that P2 is a non-enveloped phage (Fig. S5). Overall, these results suggest that the stability of P2 is relatively robust, facilitating its potential for storage and application.

### Reduced drug resistance and virulence of phage- resistant strains

**Fig 5:**
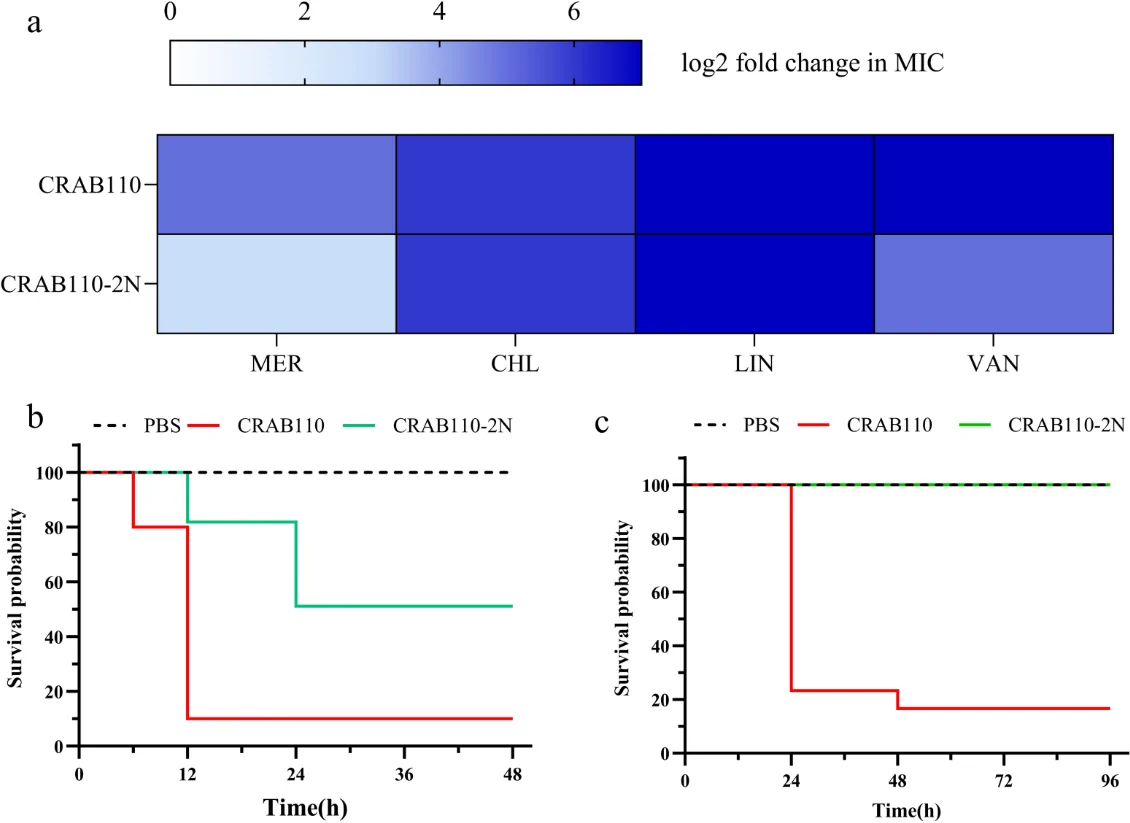
CRAB110-2N antimicrobial susceptibility and virulence assessment. (**a**) The antimicrobial susceptibility testing of CRAB110-2N demonstrated a increased sensitivity to MER and vancomycin, with MER shifting from resistant to intermediate sensitivity. (**b**) Utilizing *G. mellonella* larvae as an infection model, we evaluated the virulence of the target strain. The results indicated a significant reduction in the virulence of CRAB110-2N (*P < 0.05). (**c**) In a mouse virulence assay, we further assessed the pathogenicity of the strain, revealing a highly significant reduction in the virulence of CRAB110-2N (****P < 0.0001).

Following phage treatment of CRAB110, a bacterial strain resistant to P2 was isolated and termed CRAB110-2N. Through antimicrobial susceptibility testing of isolated phage-resistant strains, we found that the MIC of MER increased from 32 to 8 μg/mL, while the MIC of vancomycin increased from greater than 128 to 32 μg/mL (Fig. 5a). Additionally, assessment of the virulence of the resistant strains revealed a significant reduction in virulence in both the *G. mellonella* larvae and mice infection models (Fig. 5b and c). These findings provide a crucial theoretical basis for the combined use of phages and antibiotics.

### P2 Combined with MER to eliminate CRAB110

#### P2 Combined with MER to eliminate CRAB110 *in vitro*

**Fig 6:**
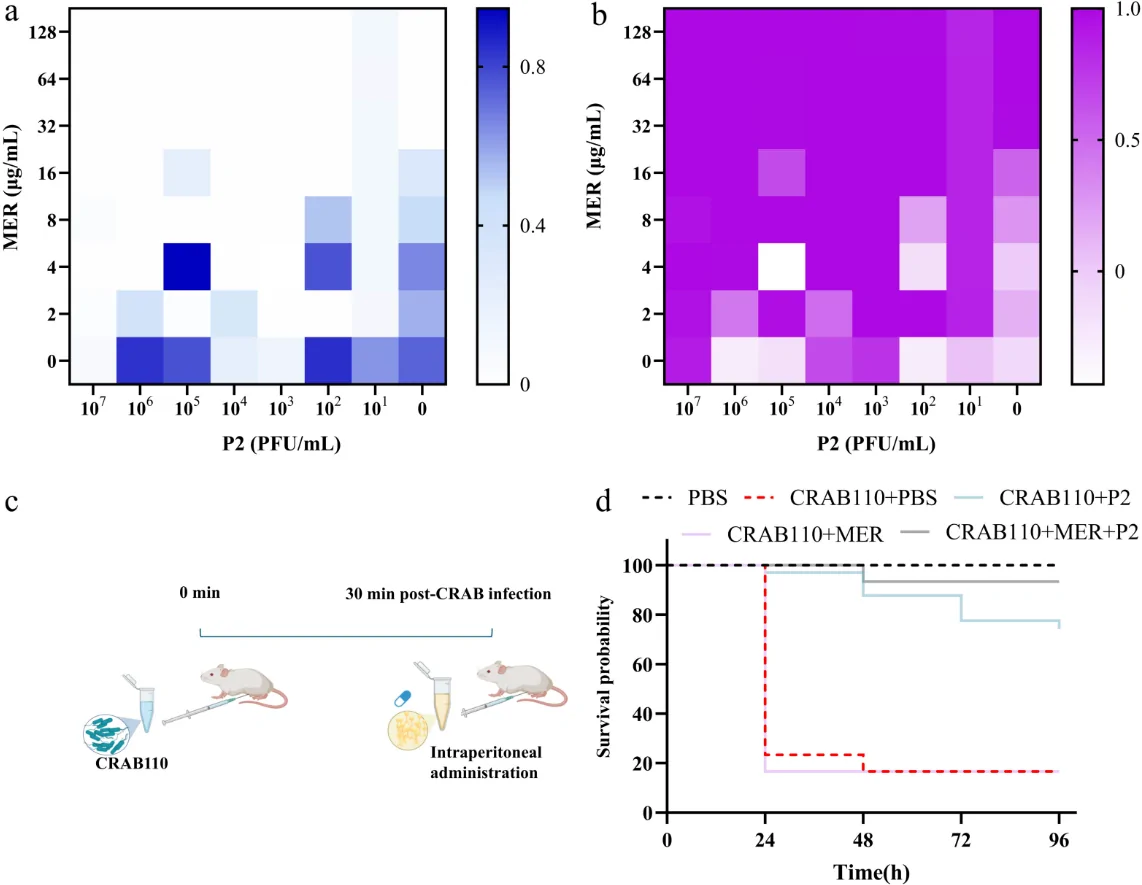
The combination therapy of P2 with MER exhibits synergistic therapeutic effects in mice infected with CRAB110. The synergogram of MER was evaluated across a concentration range of 0 to 128 μg/mL (rows) and P2 at titers from 0 to 10^7^ PFU/mL (columns). (**a**) The most significant reduction in the MIC of MER was observed when P2, at a titer of 1 × 10^7^ PFU/mL, was combined with MER at a concentration of 2 μg/mL. (**b**) Eighteen hours post-treatment, this combination achieved a 99% eradication rate of bacteria against CRAB110. (**c**) Experimental procedure: following intraperitoneal infection with CRAB110, mice were administered various intervention treatments through intraperitoneal injection 30 minutes post-infection. (**d**) Following the establishment of the infected mouse model, each group was administered either PBS, P2, MER, or the MER+P2 combination therapy, with survival rates monitored over a period of 96 hours. The results indicated that, compared to the control group, P2 monotherapy significantly enhanced the survival rate (****P < 0.0001). Furthermore, the MER+P2 combination therapy demonstrated the highest efficacy, with survival rates not only significantly greater than those of the control group (****P < 0.0001) but also markedly superior to those of the P2 monotherapy group (*P < 0.05). This finding underscores the synergistic antibacterial effects observed between the two agents.

The bactericidal efficacy of the combinatorial therapy was evaluated through the co-administration of P2 and MER at concentrations ranging from 0 to 128 μg/mL over an 18-hour incubation period. Quantitative analysis demonstrated a significant enhancement of antimicrobial activity. In the presence of the phage, the MIC of MER increased from 32 to 2 μg/mL (Fig. 6a), while a 99% pathogen clearance rate was achieved (Fig. 6b). Notably, specific phage-antibiotic ratios unexpectedly promoted bacterial proliferation, suggesting concentration-dependent bidirectional effects.

#### P2 combined with MER to eliminate CRAB110 *in vivo*

Thirty minutes after establishing the CRAB110 infection model in mice, the animals were grouped and treated with phage, MER, or a combination of both phage and MER. Continuous observation and recording of survival rates revealed that the phage monotherapy group demonstrated favorable therapeutic effects, significantly improving mouse survival rates. In contrast, the MER monotherapy group exhibited mortality rates comparable to those of the infected control group, with no apparent protective effect observed. Notably, the combination therapy group, which received both phage and MER, displayed the most favorable therapeutic outcome, with significantly higher survival rates than either monotherapy group (Fig. 6d). These results indicate that the combined use of phage and antibiotics has a synergistic effect in enhancing the treatment of CRAB110 infections.

### Safety evaluation of P2

After injecting P2 into *G. mellonella* larvae and mice, continuous observation indicated that none of the test subjects exhibited mortality or adverse reactions. Specifically, there were no instances of mortality or body surface darkening in the *G. mellonella* larvae model; similarly, the mouse model showed no mortality or abnormal behavior (Fig. S6). These results demonstrate that P2 has a favorable safety profile in both models.

### The CPS synthesis gene *ugd* mediates CRAB susceptibility to P2

**Fig 7:**
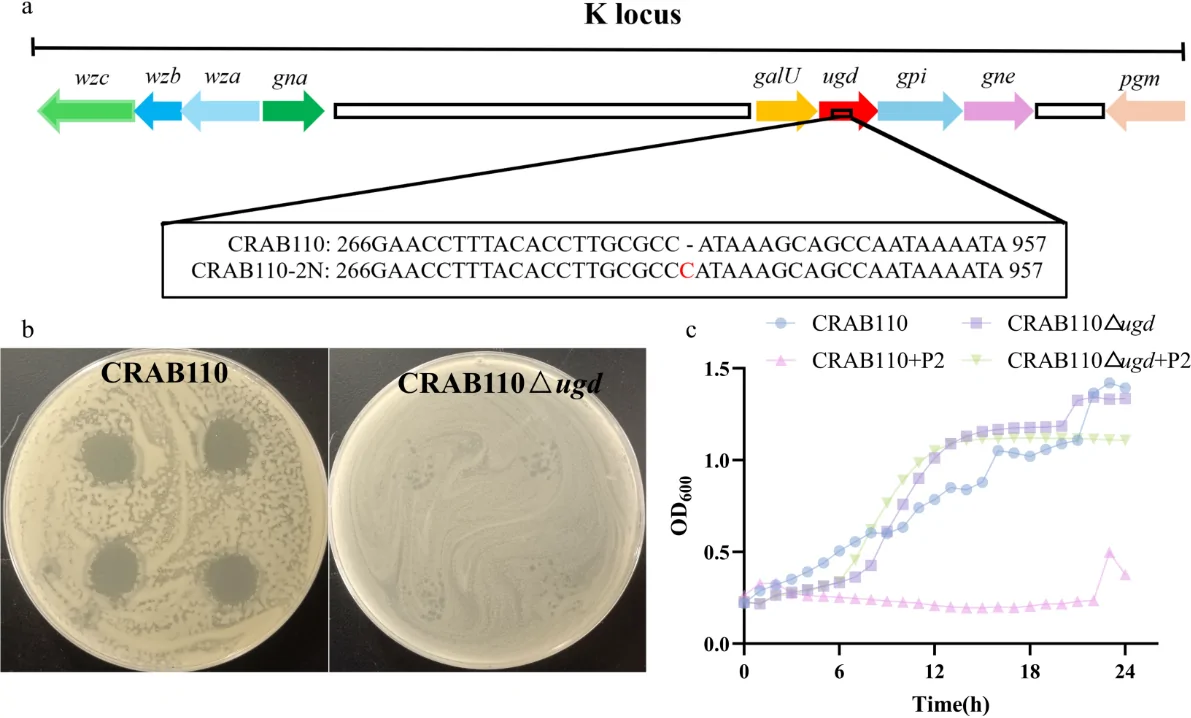
The *ugd*-mediated resistance of CRAB110 to P2. (**a**) The schematic diagram depicts CPS synthesis gene cluster in *A. baumannii*, with genes color-coded according to their predicted functions. A single cytosine (**c**) insertion mutation was identified in *ugd* of CRAB110-2N. (**b–c**) The effects of *ugd* on the lytic activity of P2 are presented. (**b**) Large, clear plaques were observed on the CRAB110 bacterial lawn, while only a few tiny plaques were detected on the CRAB110Δ*ugd* lawn. (**c**) The growth curve indicates that the addition of P2 to CRAB110Δ*ugd* does not yield a significant bactericidal effect.

A whole-genome comparative analysis of the isolated resistant strain CRAB110-2N revealed a mutation within the *ugd* locus, which encodes a crucial enzyme involved in the biosynthesis of capsular polysaccharide (CPS). The insertion of a cytosine (C) resulted in a frameshift mutation (Fig. 7a), thereby disrupting the open reading frame. Following the knockout of the *ugd* gene, the CRAB110Δ*ugd* strain exhibited significantly reduced sensitivity to P2. In comparison to CRAB110, the ability of CRAB110Δ*ugd* to form plaques was notably diminished (Fig. 7b). A quantitative assessment of bactericidal efficacy, conducted through area under the curve (AUC) analysis, indicated that P2 achieved an approximate 60% killing rate against CRAB110, while its bactericidal effect on CRAB110Δ*ugd* was only about 2% (Fig. 7c). This genetic alteration is speculated to impair CPS biosynthesis, leading to structural modifications that consequently reduce sensitivity to P2.

### Effect of QS gene *abaI* on CRAB susceptibility to P2

**Fig 8:**
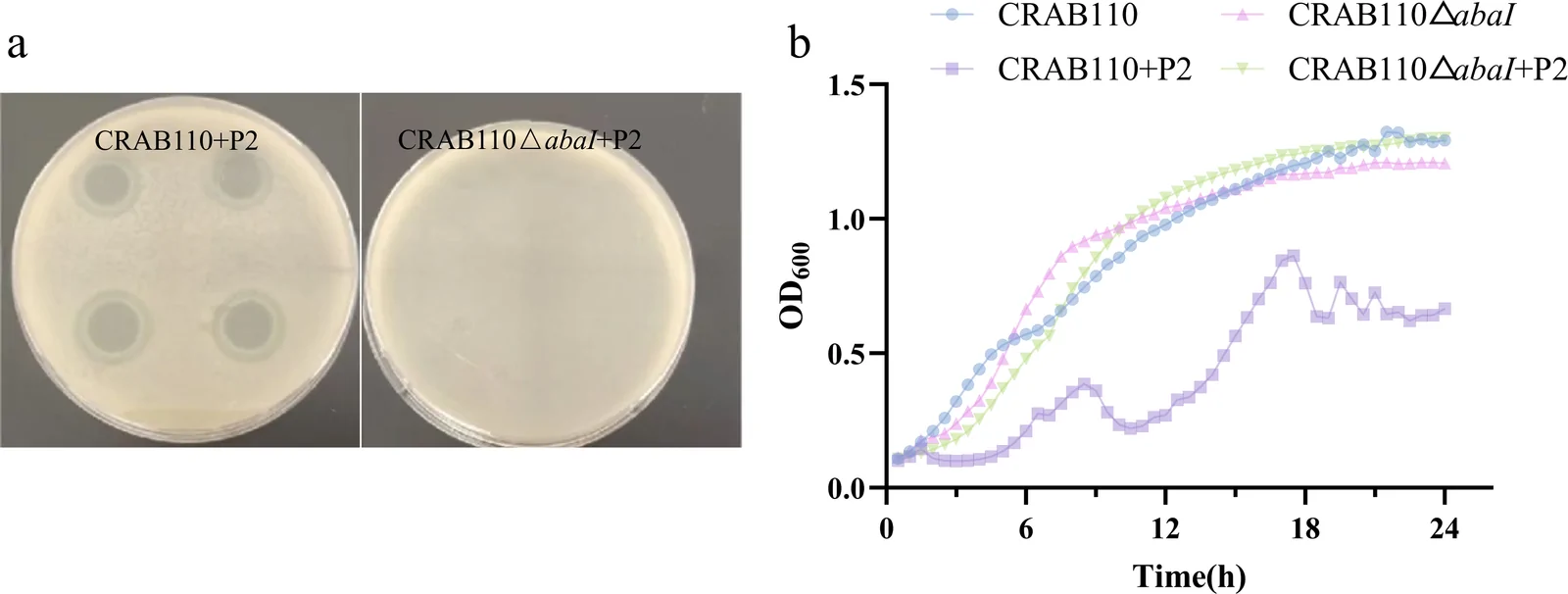
The QS gene *abaI* is associated with P2 susceptibility. (**a**) No plaque formation was observed on the plates of the CRAB110Δ*abaI*. (**b**) The bactericidal curve demonstrated an absence of significant lytic activity.

CRAB110Δ*abaI* exhibited a significantly reduced production of capsular polysaccharide compared to the wild-type CRAB110 (Zhilang Qiu). Standardized sensitivity assays confirmed the potent lytic activity of P2 against CRAB110, while no plaque formation was observed on CRAB110Δ*abaI* lawns (Fig. 8a). Quantitative evaluation of bactericidal efficacy, as assessed through AUC analysis, revealed a killing rate of 53.36% for P2 against CRAB110, contrasting sharply with the negligible efficacy of 0.40% against CRAB110Δ*abaI* (Fig. 8b). Collectively, these data demonstrate that P2 exhibits virtually no lytic susceptibility toward the capsule-deficient CRAB110Δ*abaI*.

## DISCUSSION

CRAB is a notorious pathogenic bacterium commonly found in hospital settings, and its detection has been on the rise in recent years. Phages, which are viruses that specifically target and kill bacteria, are gaining increasing attention as a potential treatment for diseases caused by drug-resistant bacteria. Generally, strains with high virulence exhibit relatively low levels of drug resistance, whereas highly drug-resistant strains tend to have lower virulence (16, 59).

Through the use of *G. mellonella* larvae and a mouse model, we assessed the virulence differences among 199 strains of CRAB, identifying the highly virulent CRAB110. Following infection of mice, the mortality rate reached 100% within 54 hours. Subsequently, we performed transcriptomic analysis on lung tissues infected with the most virulent CRAB110 and the least virulent CRAB55. The analysis indicated that the enhanced virulence of CRAB110 may be related to inflammation, with pro-inflammatory factors, such as *CCL2, TNF*, and *IL-6,* being involved (60–62). The overexpression of *TNF* can promote inflammatory responses (63). Notably, we enriched the *IL-17* signaling pathway, as *IL-17* is a pro-inflammatory cytokine (64, 65). Research has shown that *IL-17* promotes *A. baumannii* infection in the lungs of mice by inhibiting neutrophil phagocytosis during early infection stages (66). Collectively, these findings indicate a confluence of antibiotic resistance and virulence. The highly virulent CRAB poses a severe threat to public health, as its complex mechanisms of resistance and virulence exacerbate infection severity, leading to shorter host survival times and complicating clinical treatment.

This study confirms that P2 is an effective therapeutic agent against highly virulent CRAB, offering a novel strategy to combat such infections. Numerous studies have demonstrated that phage therapy can effectively reverse antibiotic resistance and diminish the pathogenicity of bacterial infections (7, 24, 25). Clinical research has confirmed that phage treatment of *Pseudomonas aeruginosa* and *Klebsiella pneumoniae* results in a certain degree of reduction in both their virulence and antibiotic susceptibility (67–70). In the present study, P2 treatment of CRAB110 similarly reduced the resistance and virulence of mutant strains, indicating that phages can be used in combination with antibiotics. When bacteria encounter phage attacks, they experience significant selective pressure that compels them to reallocate resources to mitigate the survival threat posed by phages (71, 72). To resist phages, bacteria may lose or modify certain characteristics, thereby enhancing their chances of survival. Such adaptive changes can lead to the downregulation of bacterial efflux pump expression, which subsequently affects their susceptibility to drugs (73). Furthermore, the MexAB-OprM efflux pump may modify its drug resistance in response to phage adsorption (74). Since some phages recognize bacterial capsular polysaccharides as their receptors, bacteria can evade phage detection by losing or altering their capsular polysaccharide structures, which may potentially reduce their virulence (7). Chantal Weissfuss et al (75). demonstrated that the combined phage-MER therapy exhibited superior bactericidal effects compared to monotherapy . In this study, the combination of P2 and MER demonstrated superior therapeutic effects compared to phage treatment alone, both *in vivo* and *in vitro*. This finding provides a theoretical basis for the clinical application of phage-antibiotic combinations.

The molecular recognition of *A. baumannii* by phages involves ligand-receptor interactions with surface-exposed macromolecular complexes, including CPSs, lipopolysaccharide structures, and outer membrane porins (4, 76). Significantly, the structurally heterogeneous CPSs, particularly those encoded by the K locus, serve as the primary interface for phage recognition, which determines host tropism (7). P2 exhibits strain-specific bacteriolytic activity, selectively targeting the ST2-KL34 and ST2-KL2 lineages. This limitation in host range aligns with the receptor recognition model established by Gordillo Altamirano et al., which posits that *A. baumannii*-infecting phages ΦFG02 and ΦCO01 utilize capsular polysaccharides as their primary receptors for adsorption (77). The *ugd* is a crucial component of the biosynthetic mechanism for capsular polysaccharides and has been functionally linked to pathways associated with phage susceptibility (11, 78, 79). The study conducted by Koncz et al. demonstrated that a mutation in the *ugd* gene of *A. baumannii* strain A71-1 conferred resistance to phages (7). CRAB110-2N mutant revealed a cytosine insertion in the *ugd* coding sequence. Subsequent experiments utilizing the constructed CRABΔ*ugd* demonstrated that the mutant exhibited significantly reduced sensitivity to P2 when compared to the wild-type strain. Numerous studies have confirmed that QS plays a regulatory role during phage infection. Our findings indicate that the QS gene *abaI* in CRAB110 is associated with phage sensitivity. Preliminary experiments showed that the deletion of the *abaI* gene resulted in a marked reduction in CPS thickness. Additionally, transcriptome analysis revealed significant downregulation of genes related to CPS synthesis (*wza, wzb, wzc*) (Zhilang Qiu). Therefore, we speculate that *abaI* may decrease susceptibility to phages by influencing the synthesis of CPSs.

P2 exhibits host specificity toward *A. baumannii* ST2-KL34 and ST2-KL2. Among these, ST2-KL2 is part of the internationally prevalent transmission lineage, whereas ST2-KL34 represents a non-epidemic variant. The geographical distribution of CRAB strains may limit the therapeutic applicability of this phage. Considering this regional epidemiological characteristic, it is crucial to develop phage cocktail formulations to broaden the treatment spectrum. Pioneering research by Mihály Koncz et al. demonstrated that the HSFPh phage cocktail has significant bactericidal effects against the ST2-KL3 lineage (7). Furthermore, QS is linked to P2 susceptibility, although the specific molecular mechanisms remain unclear. Deciphering this mechanism will be a key focus of future research.

### Conclusion

In this study, we identified a highly virulent strain, CRAB110, from a collection of 199 clinically isolated CRAB strains. The enhanced virulence of CRAB110 may be attributed to the activation of inflammatory responses. Utilizing CRAB110 as the host bacterium, we successfully isolated four phages, among which P2 demonstrated a broad host range, strong lytic activity, and good stability, and was classified as a novel phage. We also isolated a P2-resistant strain, CRAB110-2N, which exhibited reduced drug resistance and virulence upon biological characterization. *In vitro* experiments revealed that the combination of P2 and MER significantly improved bacterial clearance efficacy. Furthermore, *in vivo* therapeutic studies in mice confirmed that the combination of phage therapy and antibiotics yields optimal treatment outcomes. Safety experiments conducted *in vivo* indicated that P2 possesses favorable biosafety. Notably, the *ugd* gene in the CRAB110-2N strain underwent mutation, and the constructed CRABΔ*ugd* strain displayed significantly reduced sensitivity to P2. Additionally, we identified that the QS gene *abaI* may be linked to phage sensitivity. In summary, this study provides novel insights into the prevention and control of highly virulent CRAB infections and proposes potential therapeutic strategies.

## Data Availability

The GenBank accession number for the complete genome sequence of the Acinetobacter baumannii strain CRAB110-2N is JBVPYW000000000. Additionally, the GenBank accession number for the genome sequence of the Acinetobacter baumannii P2 is PZ320812. Furthermore, the accession number for the transcriptome data of lung tissue from mice infected with Acinetobacter baumannii strains CRAB110 and CRAB55 is PRJNA1457019. The genome sequence of the Acinetobacter baumannii strain CRAB110 (submission ID: SUB16139142) has been successfully submitted to GenBank.

## SUPPLEMENTAL MATERIAL

Supplemental figure legends spectrum.03962-25-s0001.docx Observe the signs of CRAB110-infected mice, conduct lung scanning electron microscopy, and detect some biological characteristics of P2.

Figure S1 spectrum.03962-25-s0002.tiff Phenotypic differences between mice infected with wild-type CRAB110 and the uninfected group. (a, c, e) Mice in the uninfected group exhibit smooth and shiny fur, normal activity levels, and a healthy mental state; (b, d, f) Mice in the CRAB110 infection group display disheveled fur, lethargy, eye inflammation, and characteristicother distinctive pathological features.

Figure S2 spectrum.03962-25-s0003.tif Observation of lung tissue via scanning electron microscopy post-CRAB110 infection. (a) In the cyclophosphamide group, the lung tissue of mice displayed a complete and smooth cavity structure, with no apparent structural damage or bacterial colonization (×2900, inset ×27500). (b) Conversely, in the CRAB110 infection group, the lung structure of mice was significantly compromised, with a considerable number of bacteria colonizing the cavities, resulting in cavity collapse. Numerous bacteria were distinctly observed at the location indicated by the white arrow (×2900, inset ×27500).

Figure S3 spectrum.03962-25-s0004.tif Phylogenetic tree construction of P2. (a) A circular phylogenetic tree of Phages was constructed based on whole-proteome similarity, with P2 indicated by a red star. (b) A rectangular phylogenetic tree, representing a zoomed-in subset of the *A. baumannii* family from the circular tree, highlights P2 (marked by a red star) and its closest relatives.

Figure S4 spectrum.03962-25-s0005.tiff P2 whole-genome functional annotation. A circular genome map is presented, revealing a genome size of 45,509 base pairs (bp). The circles in the map are arranged from the innermost to the outermost layer: the GC skew plot, the G + C% content, and the open reading frames (ORFs) transcribed in either the clockwise or counter-clockwise directions, represented by differently colored arrows according to their respective functions.

Figure S5 spectrum.03962-25-s0006.tif Illustrates the biological characteristics of P2, encompassing its host range, multiplicity of infection (MOI), and one-step growth curve.

Figure S6 spectrum.03962-25-s0007.tif Safety assessment of P2 in *G. mellonella* and mice. The Kaplan-Meier curves show: illustrate that (a) Iinfection with P2 did not causeresult in mortality in *G. mellonella larvae*; (b) Isimilarly, infection with P2 also did not causelead to mortality in mice.

Table S1 spectrum.03962-25-s0008.xlsx Assessment of virulence in 199 carbapenem-resistant *Acinetobacter baumannii* (CRAB) strains using the *Galleria mellonella* larvae infection model.

Table S2 spectrum.03962-25-s0009.xlsx DESeq2-based analysis of differentially expressed protein-coding genes in lung tissues of mice infected with CRAB110 vs. CRAB55.

ASM does not own the copyrights to Supplemental Material that may be linked to, or accessed through, an article. The authors have granted ASM a non-exclusive, world-wide license to publish the Supplemental Material files. Please contact the corresponding author directly for reuse.
