# Supplementary material for: From screening to treatment: in vitro and in vivo efficacy of phage-meropenem combination against highly virulent Carbapenem-resistant Acinetobacter baumannii
Source: Microbiol Spectr. 2026 Jun 15;14(7):e03962-25. doi: 10.1128/spectrum.03962-25 (PMC13340115; doi:10.1128/spectrum.03962-25)
Supplement: Supplemental figure legends — Observe the signs of CRAB110-infected mice, conduct lung scanning electron microscopy, and detect some biological characteristics of P2. [file spectrum.03962-25-s0001.docx]

Fig. S1 Morphological observation of the infected group and the normal saline group.a, c, e for physiological saline group, b, d, f for the infection group.

Fig. S2 Observation of lung tissue via scanning electron microscopy post-CRAB110 infection. (a) Cyclophosphamide group × 2900 ( ×27500, illustration). (b) CRAB110 infection group × 2900 (×27500, illustration).

Fig. S3 (a) A circular phylogenetic tree of Phages was constructed based on whole-proteome similarity, with P2 indicated by a red star. (b) A rectangular phylogenetic tree, representing a zoomed-in subset of the *A. baumannii* family from the circular tree, highlights P2 (marked by a red star) and its closest relatives.

Fig. S4 P2 Whole-genome functional annotation. A circular genome map is presented, revealing a genome size of 45,509 base pairs (bp). The circles in the map are arranged from the innermost to the outermost layer: the GC skew plot, the G + C% content, and the open reading frames (ORFs) transcribed in either the clockwise or counter-clockwise directions, represented by differently colored arrows according to their respective functions.

Fig. S5 illustrates the biological characteristics of P2, encompassing its host range, multiplicity of infection (MOI), and one-step growth curve. (a) The optimal MOI for P2 was determined to be 0.01. (b) The one-step growth curve of P2 is depicted. (c) The thermal stability of P2 was assessed by incubating it in LB medium at various temperatures for 30 minutes, demonstrating that the phage maintained viability across a temperature range of 10 to 60 ℃. (d) The pH stability of P2 was evaluated, revealing high stability within a pH range of 5 to 9. (e) Additionally, the stability of P2 in the presence of chloroform was investigated.

Fig. S6 The safety assessment of P2 on *G. mellonella* and mice. (a) Kaplan-Meier curves demonstrate that a significantly larger number of *G. mellonella* larvae survive the infection with P2. (b) Kaplan-Meier curves illustrate that a considerably greater number of mice survive the infection with P2.
