# Supplementary figures and images for: From screening to treatment: in vitro and in vivo efficacy of phage-meropenem combination against highly virulent Carbapenem-resistant Acinetobacter baumannii
Source: Microbiol Spectr. 2026 Jun 15;14(7):e03962-25. doi: 10.1128/spectrum.03962-25 (PMC13340115; doi:10.1128/spectrum.03962-25)

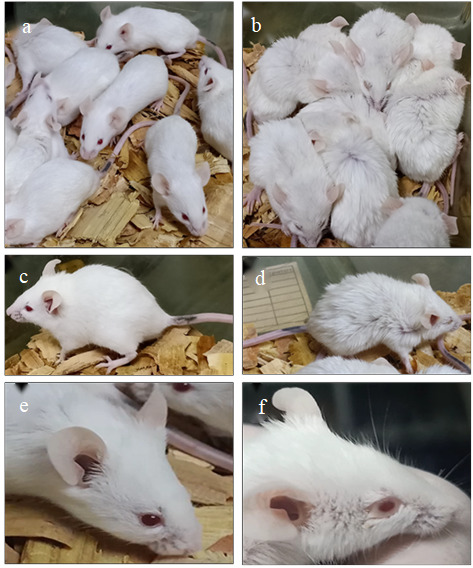

Supplement: Figure S1 — Phenotypic differences between mice infected with wild-type CRAB110 and the uninfected group. (a, c, e) Mice in the uninfected group exhibit smooth and shiny fur, normal activity levels, and a healthy mental state; (b, d, f) Mice in the CRAB110 infection group display disheveled fur, lethargy, eye inflammation, and characteristicother distinctive pathological features. [file spectrum.03962-25-s0002.tiff]

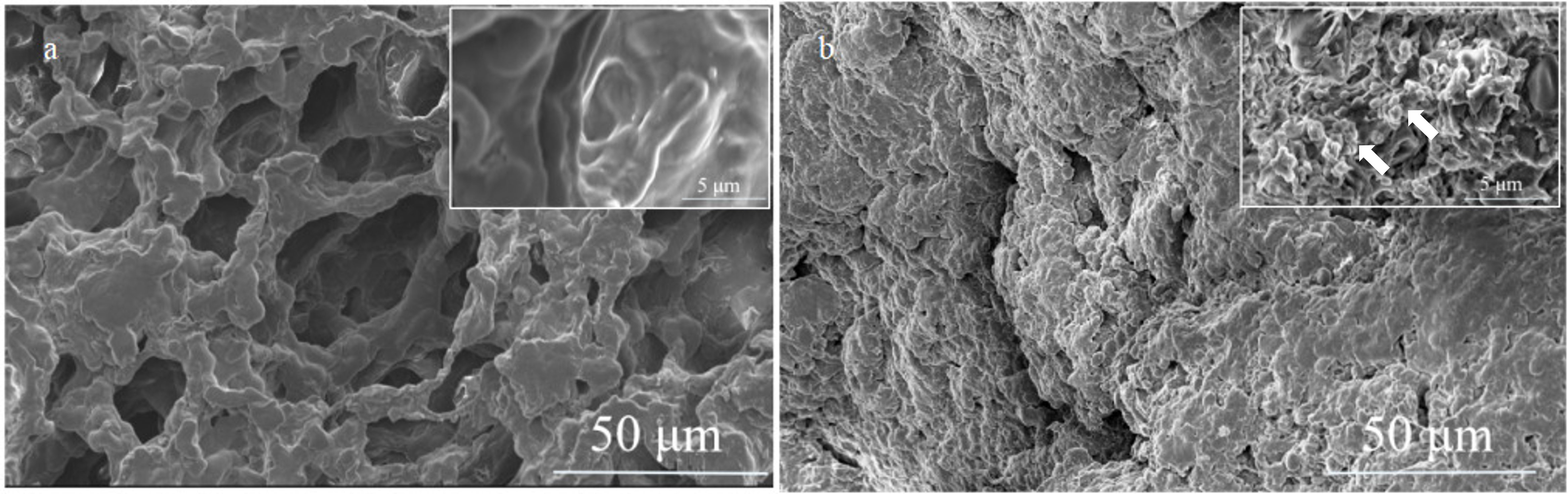

Supplement: Figure S2 — Observation of lung tissue via scanning electron microscopy post-CRAB110 infection. (a) In the cyclophosphamide group, the lung tissue of mice displayed a complete and smooth cavity structure, with no apparent structural damage or bacterial colonization (×2900, inset ×27500). (b) Conversely, in the CRAB110 infection group, the lung structure of mice was significantly compromised, with a considerable number of bacteria colonizing the cavities, resulting in cavity collapse. Numerous bacteria were distinctly observed at the location indicated by the white arrow (×2900, inset ×27500). [file spectrum.03962-25-s0003.tif]

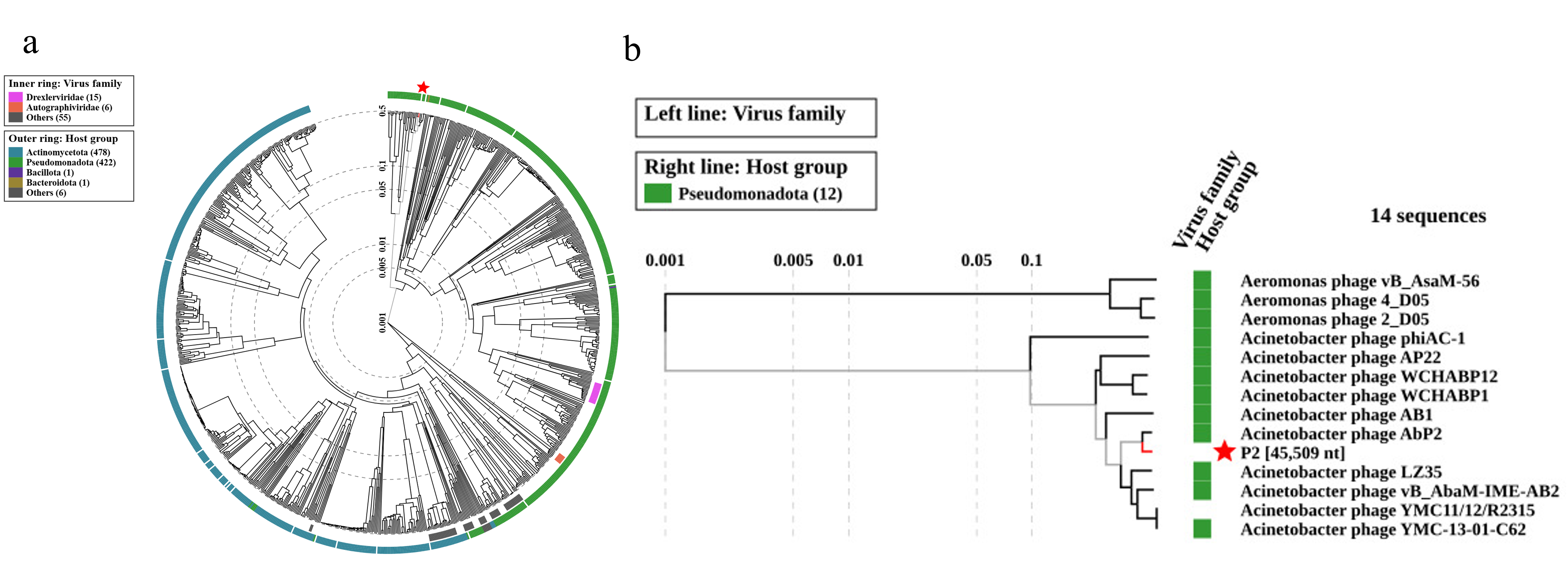

Supplement: Figure S3 — Phylogenetic tree construction of P2. (a) A circular phylogenetic tree of Phages was constructed based on whole-proteome similarity, with P2 indicated by a red star. (b) A rectangular phylogenetic tree, representing a zoomed-in subset of the A. baumannii family from the circular tree, highlights P2 (marked by a red star) and its closest relatives. [file spectrum.03962-25-s0004.tif]

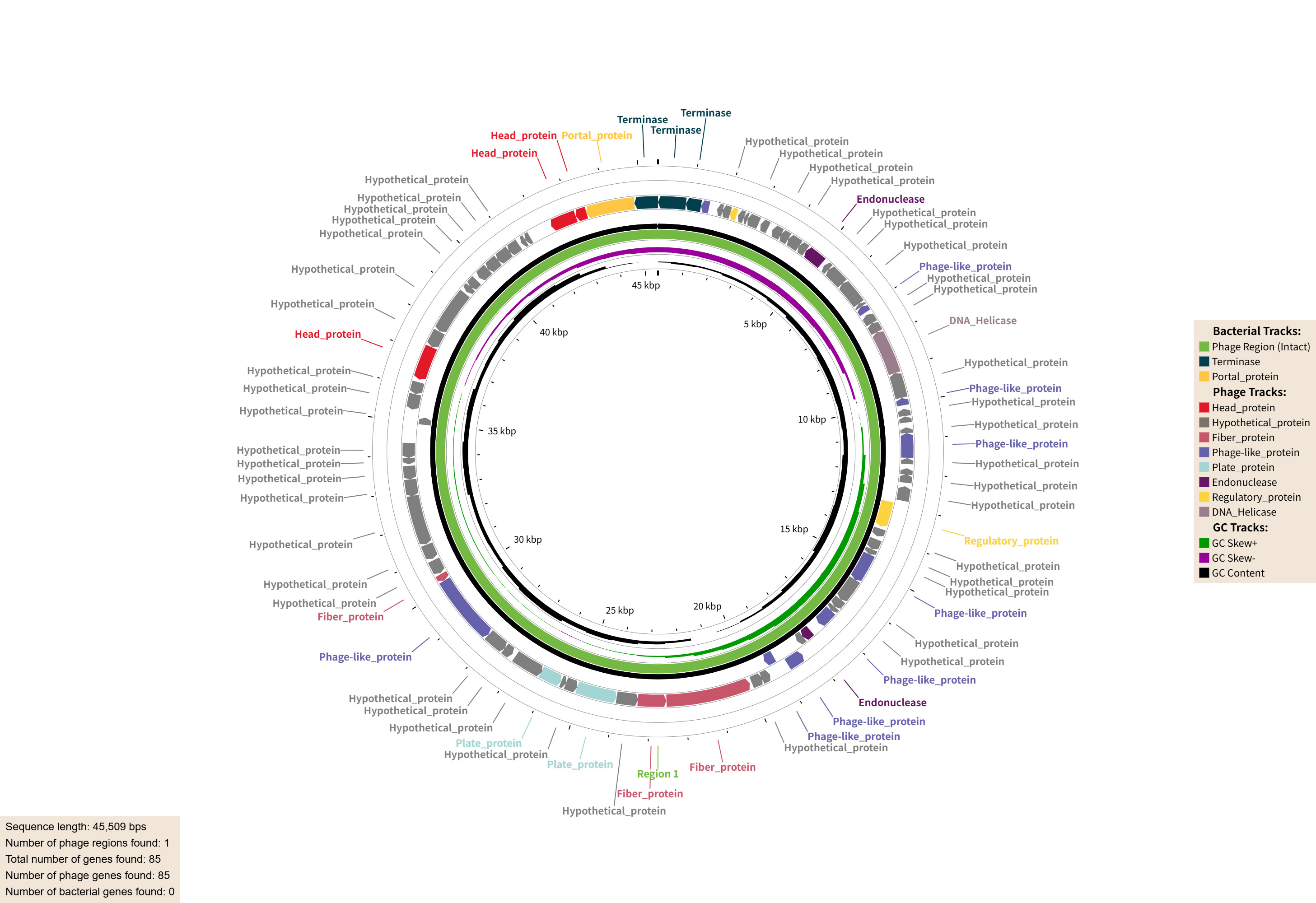

Supplement: Figure S4 — P2 whole-genome functional annotation. A circular genome map is presented, revealing a genome size of 45,509 base pairs (bp). The circles in the map are arranged from the innermost to the outermost layer: the GC skew plot, the G + C% content, and the open reading frames (ORFs) transcribed in either the clockwise or counter-clockwise directions, represented by differently colored arrows according to their respective functions. [file spectrum.03962-25-s0005.tiff]

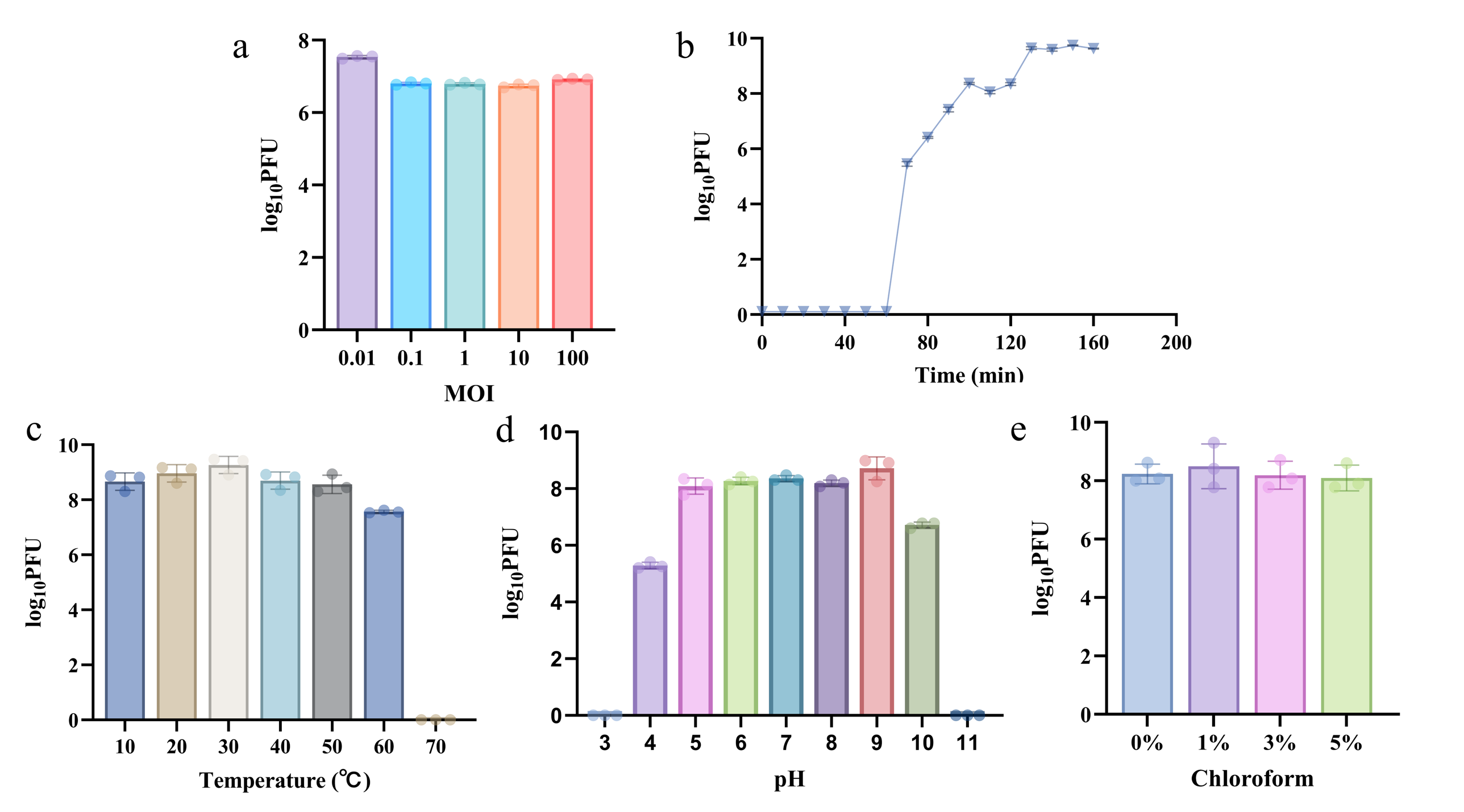

Supplement: Figure S5 — Illustrates the biological characteristics of P2, encompassing its host range, multiplicity of infection (MOI), and one-step growth curve. [file spectrum.03962-25-s0006.tif]

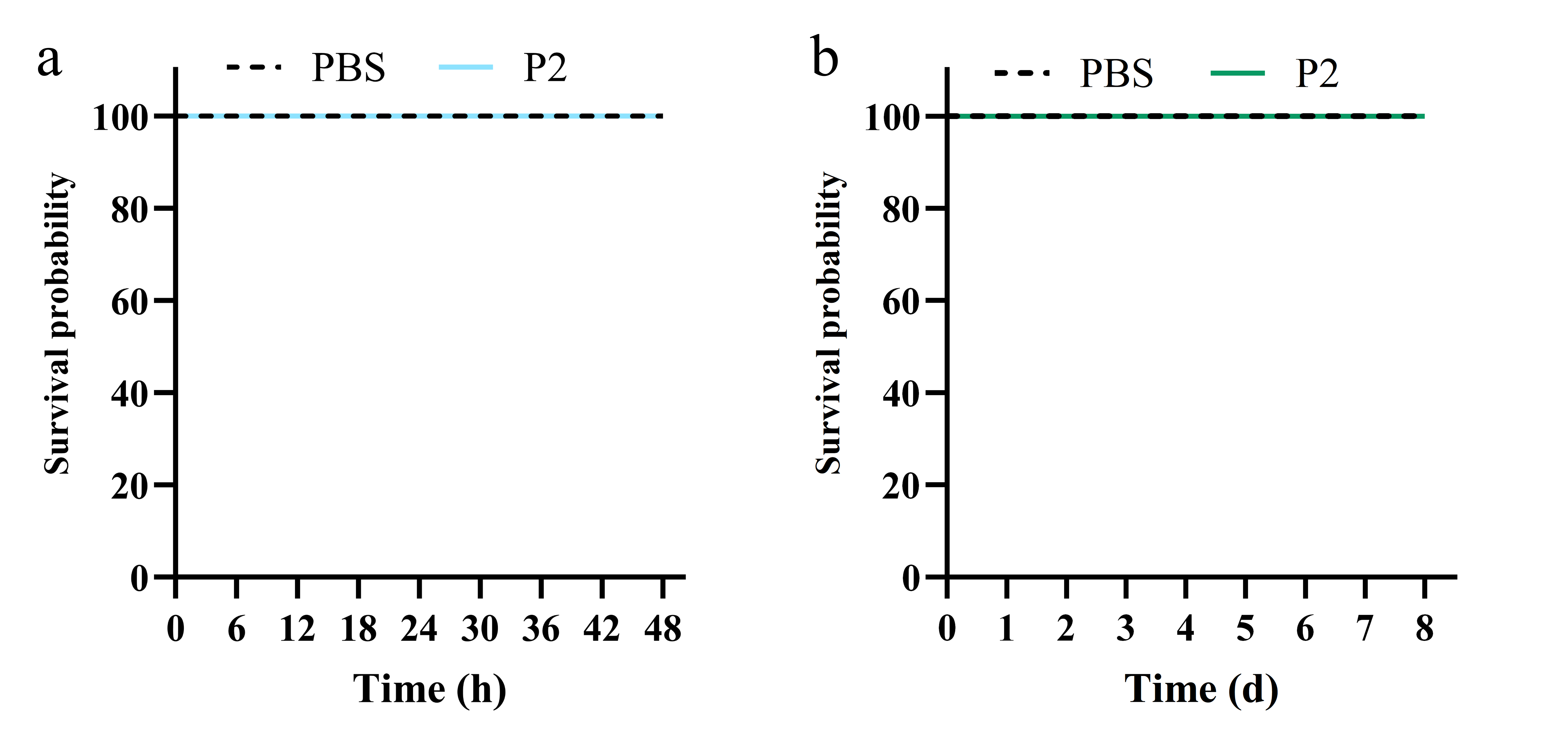

Supplement: Figure S6 — Safety assessment of P2 in G. mellonella and mice. The Kaplan-Meier curves show: illustrate that (a) Iinfection with P2 did not causeresult in mortality in G. mellonella larvae; (b) Isimilarly, infection with P2 also did not causelead to mortality in mice. [file spectrum.03962-25-s0007.tif]
